# Mind–body and art therapies impact on emotional regulation in patients with chronic diseases: a pragmatic mixed-methods randomized controlled trial

**DOI:** 10.1186/s12906-023-04173-8

**Published:** 2023-09-28

**Authors:** A. Le Rhun, P. Caillet, M. Lebeaupin, M. Duval, L. Guilmault, E. Anthoine, G. Borghi, B. Leclère, L. Moret

**Affiliations:** 1 Nantes Université, CHU Nantes, Service de Santé Publique, 44000 Nantes, France; 2 Nantes Université, CHU Nantes, INSERM, MethodS in Patients-Centered Outcomes and HEalth Research, SPHERE, 44000 Nantes, France

**Keywords:** Chronic disease, Emotional regulation, Mind–body therapies, Sensitive art therapies, Mixed methods, Clinical trial

## Abstract

**Background:**

Effective emotional regulation is recognized as essential to a good mental health of people with chronic diseases, and Mind–body and Art Therapies (MBATs) could have a positive effect on emotional regulation skills in this population. Thus, we aimed to evaluate the effect of MBATs on emotional regulation as measured by the Difficulties in Emotion Regulation Scale (DERS) questionnaire.

**Methods:**

A convergent mixed approach nested in a pragmatic superiority two arms parallel randomized controlled trial was conducted. French speaking adults with one or more chronic somatic illnesses and not suffering from a chronic psychiatric disorder unrelated to one of their chronic somatic illness were included. At inclusion, non-directive interviews were conducted, followed by an initial DERS assessment. The same combination of evaluation was implemented after 6 months of activity (T1). After inclusion, each participant was randomized within either the intervention group (G1) or the control group (G2) following a controlled wait-list design by use of a pregenerated randomization list. Staff and patient were blinded to this list until the initial evaluation was completed, after which the trial was conducted in an open-label fashion. Participants chose 2 mediations: one creativity-focused (art-therapy, writing workshop, theatre of life, vocal workshop) and one mind–body-focused (mindfulness meditation, Pilates, shiatsu, ayurvedic massages). G1 started their mediations immediately after inclusion, while G2 started 6 months later. Primary outcome was the change in means at 6 months in the overall DERS score compared between each group. Non-directive interviews were carried out at the inclusion and after 6 months of MBATs. A continuous inductive analysis was carried out on gathered material in G1 to explore the participants' experiences regarding their disease and their perceived changes associated to the intervention.

**Results:**

A total of 150 patients was randomized (75 per groups) at the end of the study. At T1, 133 patients filled out the final questionnaire (67 in G1 vs 66 in G2) and 112 interviews were analysed (54 in G1 vs 58 in G2). All 150 patients were analysed (intention to treat) using a multiple imputation approach. The mean DERS score at T0 was equal to 82.8 ± 21.1 and 85.0 ± 20.2 in G1 and G2 respectively. On average, at T1, the score decreased in the G1 (Δ = -4.8, SD = 21.3) and in G2 (Δ = -0.11, SD = 17.8). The difference in decrease, however, was not statistically significant (*p* = 0.13). Qualitative analysis underlined some MBATs benefits on emotional regulation, especially on regulation strategies. No harms related to the intervention has been observed.

**Conclusions:**

This study only partially supports benefits on MBAT on emotional regulation skills enhancement in patients with chronic disease receiving MBATs, as measured by the DERS scale.

**Trial registration:**

The protocol was registered on Clinical Trials (NCT02911207).

**Supplementary Information:**

The online version contains supplementary material available at 10.1186/s12906-023-04173-8.

## Introduction

According to the World Health Organization, for the first time, a large part of the world's population can expect to live into their sixties and beyond [1]. This increase in life expectancy leads to a significant surge in chronic diseases, as ageing and chronic ill-health are strongly related [2]. In France, around 36% of the population had a treatment or a reimbursement scheme related to a chronic disease in 2020 [3]. Chronic diseases constitute a real upheaval in patients' lives: ruptured sense of security and identity, damaged self-esteem, loss of control and meaning, and emotional isolation [4]. These shifts result in a substantial deterioration of their mental well-being and quality of life (QoL), and [5, 6]. Supporting these people, who are prone to physical, mental and social frailty, is the focus of Therapeutic Patient Education (TPE), which aims to strengthen the self-care and psychosocial skills of chronically ill patients and their families [7]. Since 2010, all French people suffering from a chronic illness have been entitled by law the possibility to benefit TPE for free. A core set of psychosocial skills is at the heart of skill-based health promotion: decision making and problem solving; creative and critical thinking; effective communication and interpersonal relationship skills; self-awareness and empathy; coping with emotions and with stress [7]. Effective emotional regulation is recognized as essential to a good mental well-being and quality of life, and could also impact the physical health of people with chronic diseases [8] Improving emotional expression in several chronic diseases led to decreased distress and mood improvement [5, 6]. Emotional regulation is defined as the individual's ability to balance emotional responses, but also the ability to experience a wide range of emotions, to distinguish their nature and to accept these emotions rather than trying to repress them [9]. The efficacy of TPE is increased when patients’ emotional skills are enhanced [7].

A large panel of methods are used in TPE [10], but the use of mind–body and art therapies (MBATs) is still not widespread in France. Mind and body practices are a large and diverse group of procedures or techniques that are administered or taught by a trained practitioner or teacher. Examples include acupuncture, massage therapy, meditation, relaxation techniques, spinal manipulation, tai chi, and yoga. Art therapy is a form of psychotherapy that uses artistic creation (drawing, painting, collage, sculpture, etc.) as its main mode of expression and communication. MBATs can be used to diminish tension and stress, but also to reinforce the physiological and psychological well-being of an individual.

Art therapy experiences have showed a decrease in anxiety symptoms [11] and writing could have the same positive effects on people with cancer [12, 13] or chronic mental illness [14]. For example, drama is used in psychiatry as a mediator in interpersonal relationships and socio-emotional adjustments [15]. A systematic review showed a positive effect of arts interventions such as dance, singing, music and theatre on quality of life in patients suffering from Parkinson disease [16]. Singing is believed to have an impact on the well-being of patients with mental illness [17].

Regarding mind–body therapies, meditation and Pilates have shown interesting effects on chronically ill patients, especially in reducing anxiety and depressive symptoms [18, 19]. Meditation could induce self-regulatory behaviours and increase experiences of positive emotional states [20] and significantly decreases anxiety and depression in chronic patients [19, 21]. A few studies on shiatsu have shown a decrease in behavioural disorders in patients with dementia, especially agitation [22].

However, data regarding the efficiency of MBATs on emotional regulation is lacking [23]. Our study aims to provide elements for assessing the relevance of proposing support programs (creative and psycho-corporal mediation) that complement existing ETP programs, as they potentiate the beneficial effects on mental health—programs that have not been defined following the HPST law, or to reflect on the relevance of existing ETP programs integrating these psychosocial support practices into ETP. In fact, some teams, convinced of their benefits, are already doing so in practice (art therapy, mindfulness meditation, for example). In our case, the « Promotion Education Santé» department of Nantes University Hospital created in 2012 an association entitled « la Fabrique Créative de Santé (FCS)» with the aim of providing psychosocial support to patients with chronic diseases. Through this association and in addition to the therapeutic education programs, patients with chronic diseases could attend free creative and mind–body therapies, which they could choose without any constraints. This supportive program was delivered by professionals, with the aim to strengthen the usual therapeutic care -often hospital-based- and to promote health.

More specifically, our study aimed to 1) quantitatively measure the effect of MBATs on emotional regulation in chronic patients and 2) to describe qualitatively the emotional change experienced by patients, and more specifically their feelings, cognitions and behaviours to get a better understanding of the quantitative findings.

## Methods

**Fig. 1:**
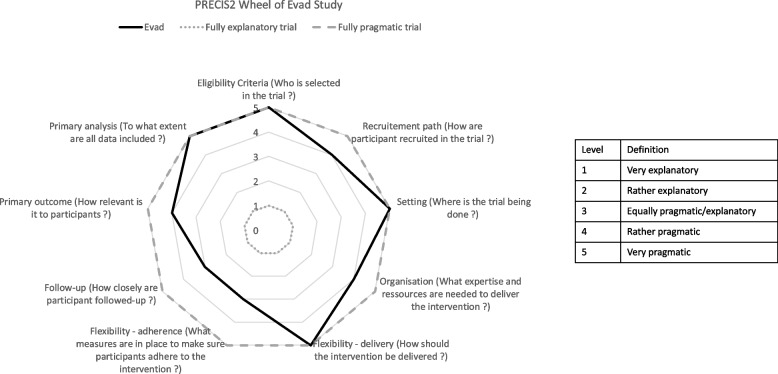
Pragmatism of the EVAD trial

We used a concurrent triangulation mixed methods design based on a 1:1 allocation ratio within a pragmatic randomized clinical trial with two parallel groups, one benefiting from the intervention immediately (G1) and the other starting the intervention six months later (G2). The degree of pragmatism in our trial is presented with the PRECIS-2 tool [24] in Fig. 1 and described in more detail in the supplementary material (see Additional file 1).

### Population

The included patients were adults with one or more chronic somatic illnesses, who had given their consent, who spoke French, who were able to carry out a creative and physical activity and who had no chronic psychiatric disorder unrelated to a chronic somatic illness, and had been offered participation in MBATs proposed within the FCS. Additionally, advertising in local press was set up to bolster recruitment. All participants had been informed by a clinical department of Nantes University Hospital or had read about the research in the local press. The inclusion period spanned from August to September 2016.

### Randomization procedure

The study used a randomized controlled wait-list design. All participants attended an initial evaluation of 1 h at inclusion (T0), which consisted of a non-directive interview focused on the patient’s experience of the disease, followed by an initial emotional regulation assessment (T0). After the initial evaluation, each participant was randomized within one of the two following groups: immediate intervention group (G1) and delayed intervention group (G2). The randomization list was created with Microsoft Excel software by the statistician, without blocking, before the investigators made the inclusions. Staff who included participants and made treatment allocations were blinded to this list until the initial evaluation was completed, after which the trial was conducted in an open-label fashion.

### Intervention

MBATS proposed by the FCS in usual care setting, as described in Table 1, constituted the components of the intervention tested in the study. Within each group, participants had to choose 2 mediations (Table 1), one being a creativity-focused mediation (art-therapy, writing workshop, theatre of life, vocal workshop) and the other a mind body-focused mediation (mindfulness meditation, Pilates, shiatsu, ayurvedic massages). This choice was made in order to profit from both the psychic and bodily dimensions of MBATs. Patients randomized into the intervention group (G1) started their mediations immediately after the end of the inclusion period (at least 10 sessions between October 2016 and February 2017). Patients in the control group (G2) started their mediations 6 months later (from February 2017 to July 2017). After 6 months of activity (T1: February 2017 for patients in G1 or July 2017 for patients in G2), a final non-directive interview exploring the changes perceived by the patients was conducted, combined with the final emotional regulation assessment. Each intervention was carried out by instructors who had signed an ethical charter, received at least 6 days' training in ETP, and all had a degree in their specialty and several years' experience in the field. All were monitored by the investigators/coordinators to ensure the quality of their intervention, and patient satisfaction was also monitored.

**Table 1:** Type of activities tested

**Body interventions**	**Description**	**Individual/collective**	**Session count**
MBSR: Mindfulness Based Stress Reduction Meditation	A mental practice which consists in focusing one’s attention on certain objects, thoughts, emotions or body part	Collective (3 to 9)	8 × 2h30 + 1 day over 3 months
Pilates	A set of physical exercises designed to strengthen core muscles and balance the body	Collective (3 to 5)	15 × 1 h over 6 months
Shiatsu	A relaxation method from Japan, based on the application of finger pressures on acupuncture points	individual	5 × 1 h over 6 months
Ayurvedic massage	A massage technique from India, considered as a body therapy	individual	5 × 1 h over 6 months
**Creative interventions**	**Description**	**Individual/collective**	**Session count**
Art-therapy	A form of psychotherapy which uses artistic creation	Collective (3 to 4)	6 × 2 h over 6 months
Writing workshop	A therapeutic approach which uses writing for introspective or creative purposes	Collective (5 to 8)	10 × 1h30 over 6 months
Theatre of Life	A therapeutic approach which promotes patients’ acceptance of their disease, through drama	Collective (4 to 6)	4,5 days over 6 months
Vocal workshop	A singing practice focused on emotions, breath and voice control	Collective (3 to 4)	7 × 1h30 over 6 months

### Quantitative emotional regulation assessment

The Difficulties in Emotion Regulation Scale (DERS), developed by Gratz & Roemer in 2004, is the Gold Standard to assess emotional regulation [9]. This self-report scale includes 36 items corresponding to 6 dimensions exploring difficulties in emotional regulation: Non-acceptance (ability to accept one's emotional response), Goals (ability to adopt goal-oriented behaviours in a negative emotional context), Impulse (ability to control oneself in a negative emotional context), Awareness (ability to monitor, value, and maximize experience of one’s emotion), Strategies (ability to implement emotional regulation strategies in a negative emotional context) and Clarity (ability to identify, distinguish and describe specific emotion).

Participants had to indicate how often each item applied to themselves, with responses ranging from 1 to 5, where 1 is almost never and 5 is almost always. The scale yields a total score (range 36 – 180), where higher scores indicate a greater degree of impairment in emotional regulation. Reverse items are recoded so that higher scores in every case indicate greater difficulties in emotional regulation. The DERS has demonstrated excellent internal consistency, with Cronbach's alpha coefficients higher than 0.90 for the overall score and > 0.80 for each of the 6 dimensions and excellent construct validity [8].

In the absence of a Metropolitan French version, we chose to adapt the DERS from the Swiss [25] and Canadian [26] French versions. The good practices recommendations from the literature concerning the process of transcultural adaptation of questionnaires [27, 28] were respected: the Canadian and Swiss French translations were compared, the divergences were discussed, and semantic and conceptual equivalences were suggested in order to achieve a finalized version of the scale in Metropolitan French. The questionnaire was then tested for face validity and for comprehension difficulties on a panel of 10 patients who had already benefited from the mediations in another setting. The questionnaire was modified according to their feedbacks and the final version was used for the experimental phase of the project.

### Quantitative analysis

The characteristics of the study population were described using descriptive statistics. The analysis was performed with an intent-to-treat approach, i.e. on all patients randomized to the initial group regardless of their participation in activities. Missing data regarding the outcome were dealt with by implementing a multiple imputation approach with a predictive mean matching method. Five datasets were generated and pooled in the analysis. Data were described with frequency or percentage for qualitative variables, and with mean and standard deviation for quantitative variables. Data from the DERS questionnaires were described by item and the overall score and sub-scores were calculated.

The comparison of groups G1 and G2 between T1 and T0 was carried out by calculating the difference in means in the overall DERS score within each group. The differences were presented with their 95% confidence interval in brackets. The statistical significance of these differences was tested with Student's t-test. Alongside the comparison of the global DERS score, we also compared with the same procedure the evolutions of each of the DERS sub-scores between groups. In order to investigate the effect of assiduity on the results, we also performed a unplanned subgroup analysis, using the same procedure as described above. This analysis was based on the comparison of the subset of patients who participated in 80% or more of the planned sessions at T1 with the control group used in the main analysis. In case of multiplicity of test, the inflation of error risk was accounted using the Benjamini Hochberg correction [29].

Considering the most pessimistic effect observed in the literature [30, 31], an alpha and beta risk fixed at 5% and 20% respectively, a total of 130 patients (65 per group) was needed to show a statistically significant mean difference of 19 (standard error: 38.17). All analyses were performed using the R software v3.6 to 4.0.

### Qualitative analysis

Non-directive interviews were carried out at the inclusion to explore the participants' experiences regarding their disease, and after 6 months of MBATs to explore the participants' perceived changes associated to the intervention. The interviews were conducted by 4 investigators trained in TPE and 5 medical residents, all of whom had benefited from a 5-day training. These in-depth interviews, which lasted for an hour, respected the rules of confidentiality and anonymity and were recorded to allow a complete verbatim transcript of the responses. The interviews focused on the two following open-ended questions: “After 6 months of MBATs, where are you on your path to live with the disease?” and “In which areas of your life have you seen changes linked to the activities you have performed?”.

A qualitative content analysis was undertaken, which involved a continuous inductive analysis carried out to establish categories that go further than the ones based on the initial hypotheses of the DERS, in order to better understand and explain changes in emotional regulation.

All interviews (*n* = 112) were audiotaped with the permission of the participants, transcribed in verbatim, and later processed independently by two analysts (a research coordinating engineer and the coordinating investigator) using a thematic analysis. In order to avoid influence from DERS classification, we chose a conventional content analysis approach to identify themes [32]. The analysis was conducted in an inductive manner to produce themes from the material.

The analysis was conducted according to the following steps: (i) the transcribed interviews were read thoroughly, in order to achieve an overview of data and approach experimental themes; (ii) units of meanings linked to emotional regulation were identified and coded; (iii) coded data was condensed and abstracted within each of the categories, which were simultaneously elaborated, and (iv) the content of each interview was organized between each category [33].

All the 112 interviews were coded in the SPHINX QUALI software. The verbatim are reported within quoting marks and in italics. Modifications made for clarity are indicated within brackets. Translation was made freely from French to English by a non-native speaker.

### Ethics and regulation

All the requirements of French Law were fully complied with. An ethics committee (Comité de Protection des Personnes – Tours – Région Centre-Ouest 1) endorsed the protocol on June 28, 2016. The protocol and the database were registered on May 12, 2016 by the French National Data Protection Committee (CNIL) according to the requirements of the declaration of conformity. The protocol was registered on Clinical Trials under the reference NCT02911207.

### Availability of data and materials

The dataset(s) supporting the conclusions of this article is(are) available upon request to the authors.

## Results

**Fig. 2:**
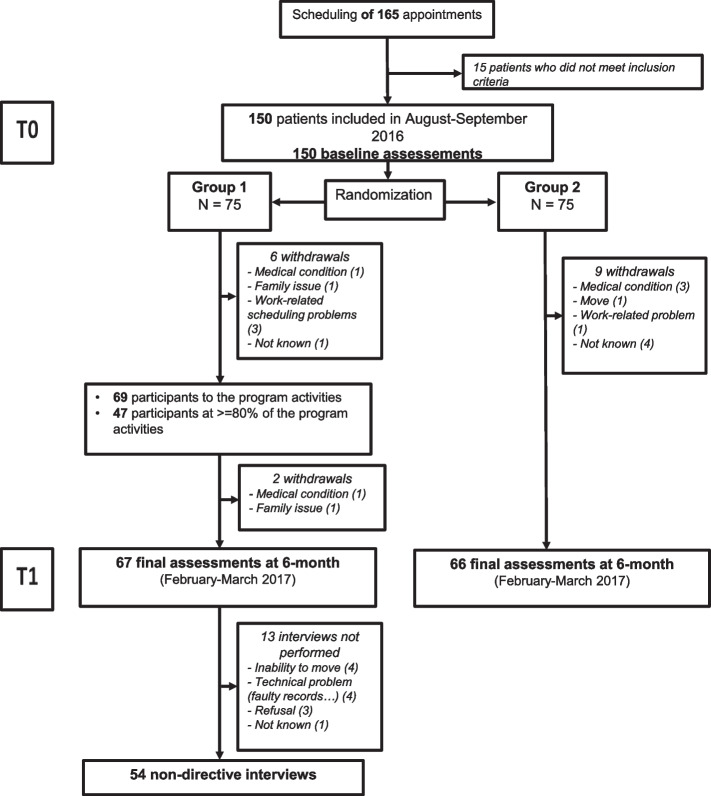
Flowchart of the study

A total of 150 patients were included at T0 (75 in G1 and 75 in G2). The flowchart of the inclusion process is displayed in Fig. 2.

Among those patients, 78% (*n* = 117) were women, mean age was 53.9 years ± 13.0 (median: 56 years; range: 20–79), 32% (*n* = 48) of the patients lived alone. Nearly three quarter (77%, *n* = 115) of them had an education level equivalent to or higher than a bachelor's degree. Regarding professional activities, 33% of the patients were retired (*n* = 50), 26% (*n* = 39) were unemployed and 41% (*n* = 61) were professionally active. Nearly 61% (*n* = 92) of the patients suffered from a single chronic somatic disease, 22% (*n* = 33) from 2 chronic diseases and about 15% (*n* = 23) from 3 or more. The median duration since diagnosis was 10 years. The most frequent pathology groups were chronic rheumatic or neurological diseases, HIV seropositivity, and gastroenterological illness. Nearly a quarter (24%, *n* = 36) of the participants had already benefited from one or more therapeutic education sessions and 60% (*n* = 90) were patients of Nantes University Hospital. All these socio-medical characteristics were comparable at T0 among groups (Table 2).

**Table 2:** Patients’ characteristics (*n* = 150) at Baseline

**Variable**	**Intervention group (*n* = 75)**	**Control group (*n* = 75)**
Age (med (range))	55 (21–76)	56 (20–79)
Women (n, %)	60 (80)	57 (76)
Higher education (bachelor degree or higher) (n, %)	58 (77)	57 (76)
Marital life (n, %)	41 (55)	42 (56)
Occupation (n, %)		
Craftsmen, merchants and entrepreneurs	2 (3)	1 (1)
Executives	6 (8)	7 (9)
Intermediate professions and employees	25 (33)	19 (25)
Workers	1 (1)	0 (0)
Retired	21 (28)	29 (39)
No occupation	20 (27)	19 (25)
Main chronic disease (n, %)		
Rheumatologic illness	14 (19)	10 (13)
Neurological illness	7 (9)	10 (13)
AIDS/HIV	6 (8)	9 (12)
Gastroenterological illness	4 (5)	9 (12)
Diabetes	5 (7)	6 (8)
Fibromyalgia	8 (11)	3 (4)
Cancer	4 (5)	7 (9)
Other	48 (64)	54 (72)
Multiple chronic diseases (n, %)	31 (41)	26 (35)
Prior experience with activities available in the intervention (n, %)	12 (16)	24 (32)
Ongoing psychological follow-up (n, %)	22 (29)	17 (23)
**Means and Standard Deviations for DERS questionnaire (Baseline)**	**Intervention group**	**Control group**
	Mean (SD)	Mean (SD)
DERS Total	82.8 (21.1)	85.0 (20.2)
DERS Non-acceptance	14.5 (5.65)	15.8 (5.8)
DERS Goals	13.9 (4.0)	14.4 (4.5)
DERS Impulse	12.7 (5.1)	12.7 (5.0)
DERS Awareness	13.7 (4.6)	13.7 (4.35)
DERS Strategy	17.5 (6.0)	17.9 (5.9)
DERS Clarity	10.1 (3.8)	10.3 (3.5)
**Means and Standard Deviations for DERS Scales at 6 months**		
DERS Total (mean, SD)	78.0 (20.0)	84.9 (21.3)
DERS Non-acceptance (mean, SD)	14.2 (5.6)	14.7 (5.5)
DERS Goals (mean, SD)	13.3 (4.6)	14.3 (4.4)
DERS Impulse (mean, SD)	11.1 (5.1)	12.5 (4.9)
DERS Awareness (mean, SD)	13.2 (4.4)	14.3 (4.9)
DERS Strategy (mean, SD)	16.3 (6.1)	18.6 (7.1)
DERS Clarity (mean, SD)	9.6 (3.3)	10.3 (3.9)

After 6 months (T1), 133 patients filled out the final questionnaire (67 and 66 patients for G1 and G2 respectively). In the intervention group, 63% (47 of 75) of the participants completed 80% or more of their activity program. There were no socio-demographic discrepancies between patients who carried out 80% and more of program activities and the others within the intervention group (Table 3). A total of 112 non-directive interviews were conducted after 6 months of MBATs activities (54 for G1 and 58 for G2 respectively).

**Table 3:** Differences between patients who carried out 80% or more of program activities and the ones who did not, within the intervention group

Adherence to the intervention (percent of planned sessions where present)	< 80% (*n* = 28)	≥ 80% (*n* = 47)	*P* value
Age (mean (SD))	53.00 (13.95)	52.70 (13.23)	0.927
Women (n, %)	21 (75.0)	42 (89.4)	0.188
Higher education (bachelor degree or higher) (n, %)	8 (28.6)	11 (23.4)	0.823
Marital life (n, %)	14 (50.0)	29 (61.7)	0.453
Occupation (n, %)			0.278
Craftsmen, merchants and entrepreneurs	1 (3.6)	1 (2.1)	
Executives	2 (7.1)	4 (8.5)	
Intermediate professions and employees	5 (17.9)	20 (42.6)	
Workers	0 (0.0)	1 (2.1)	
Retired	10 (35.7)	11 (23.4)	
No occupation	10 (35.7)	10 (21.3)	
Number of chronic diseases (mean (SD))	1.68 (0.77)	1.55 (0.88)	0.535
Prior experience with activities available in the intervention (n, %)	2 (7.1)	1 (2.2)	0.657
Ongoing psychological follow-up (n, %)	9 (33.3)	13 (27.7)	0.803

### Quantitative results

#### Evolution of the DERS score

The mean DERS score at T0 was equal to 82.8 ± 21.1 and 85.0 ± 20.2 in G1 and G2 respectively (Table 2). On average, at T1, the score decreased in the intervention group (G1) (Δ = -4.8, SD = 21.3) and diminished very slightly in the control group (G2) (Δ = -0.11, SD = 17.8). This difference, however, was not statistically significant (*p* = 0.13) (Table 4). Among patients who completed at least 80% of their activity program, the average evolution of the DERS scores was equal to -9.7 (SD = 20.0) in G1, which was significantly different when compared to the control group (G2, Δ = -0.11, SD = 17.8, *p* < 0.01). There was no statistical evidence of an association between a particular characteristic of the sample and the completion rate in the intervention group.

**Table 4:** Intra-group differences between T0 and T1 for the 2 groups, for all the participants to the program activities and for the ones from G1 who carried out 80% and more of program activities

	**All participants (*n* = 150)**					**Restricted to G1 participants who carried out > = 80% of program activities** **(unplanned subgroup analysis)**				
	**G1 (T0-T1)** ***N* = 75**		**G2 (T0-T1)** ***N* = 75**			**G1 (T0-T1)** ***N* = 47**		**G2 (T0-T1)** ***N* = 75**		
	**Mean**	**SD**	**Mean**	**SD**	***p***	**Mean**	**SD**	**Mean**	**SD**	***p***
**DERS Total**	-4.8	21.3	-0.11	17.8	0.13	-9.7	20.0	-0.11	17.8	**0.008**
**DERS** **Non-acceptance**	-0.32	6.1	-1.1	5.0	0.39	-1.4	6.2	-1.1	5.0	0.79
**DERS Goal**	-0.63	4.4	-0.14	4.0	0.46	-1.45	3.5	-0.14	4.0	0.06
**DERS Impulse**	-1.7	5.4	-0.13	4.7	0.06	-2.6	4.3	-0.13	4.7	**0.004**
**DERS Awareness**	-0.49	4.6	0.65	4.2	0.10	-1.2	4.1	0.65	4.2	0.02*
**DERS Strategy**	-1.15	7.1	0.71	5.3	0.07	-2.0	6.5	0.71	5.3	0.02*
**DERS Clarity**	-0.46	3.6	-0.02	3.7	0.47	-0.94	3.3	-0.02	3.7	0.17

^*^No statistically significant differences after accounting for tests multiplicity by use of the Benjamini–Hochberg method

#### Evolution of the DERS sub-scores

In the overall sample, none of the 6 DERS sub-scores were significantly improved by the intervention. In patients who followed 80% or more of their activity program, we observed a significant decrease in the impulse dimension in the intervention group compared to the control group (-2.6 vs -0.13, *p* = 0.004) (Table 3). The detailed description of each DERS item is presented as supplementary material (see Additional file 2).

### Qualitative analysis

The vast majority of the participants (91%, 102/112) reported an improvement in at least one aspect of their emotional regulation. The verbatim are presented in the Table 5.

**Table 5:** French verbatim excerpts and their English translation

N°	Original French	English translation	Participant’s characteristics
1	« Avant, j’étais un petit peu soupe au lait. Aujourd’hui, je le suis moins parce que je prends le temps de me poser. Parce qu’avant, les portes claquaient plus facilement ou je sautais dans ma voiture en disant « j’en ai marre, ils m’énervent tous», alors que maintenant j’arrive à mieux me gérer, contrôler, oui, à réfléchir un petit peu avant d’exploser.»	*“I used to have a short fuse. Now, it’s not as true anymore as I take the time to settle down. Because before, the doors slammed more easily or I would jump in my car and say ‘I'm fed up, they're all bugging me’, whereas now I can better manage myself, control, yes, think a little bit before exploding.”*	Participant 56: 74-year-old woman, living alone, retired Crohn's disease and ankylosing spondylitis (Meditation, writing)
2	« Pourtant je sentais dans le corps que ça titillait, mais je ne mettais pas de mots dessus. Donc maintenant, si je m’arrête, si je fais attention à ce qu’il se passe dans mon corps, eh bien je vais arriver, par le travail sur moi, à savoir ce qu’il se passe.»	*"Before, I sometimes felt in my body that something was nagging me, but I didn't put words on it. So now, if I stop, if I pay attention to what's going on in my body, then I'm going to understand, by working on myself, what's going on.”*	Participant 74: 67-year-old woman living alone, retired Breast cancer, hereditary angle-closure glaucoma, age-related macular degeneration, osteoarthritis (Meditation, art therapy)
3	« J’ai pris conscience que je fonctionne surtout pour les autres et pas assez pour moi. Je suis quelqu’un qui est trop dans l’empathie en fait avec les autres. Et c’est vraiment à mon détriment. Et là, par contre, je fais hyper gaffe.»	*""I have become aware that I function mostly for others and not enough for myself* *I am someone who is in fact too empathetic, and that's really to my detriment. And now, on the contrary, I'm being overly cautious.”*	Participant 12: 69-year-old woman living with spouse, retired Anal incontinence (Pilates, art therapy)
4	« Déjà, j’ai compris qu’il fallait accepter les émotions et les respirer. C’est normal de ressentir ce genre d’émotion, de la tristesse, de la colère et qu’il ne faut pas les garder au fond de nous parce que sinon, un jour ça ressort et puis ça fait encore plus mal. J’ai compris, avec le massage et le chant, qu’en fait, que ressentir des émotions n’était pas de la faiblesse et que la force était de les accepter et de les surmonter.» (6)	*"I understood that you have to accept your emotions and breathe them in. It's normal to feel this kind of emotion, sadness, anger, and we shouldn't keep them insides because if we do, one day it comes out and then it hurts even more. I understood, with the massage and the singing, that in fact, feeling emotions was not a weakness and that the strong thing to do was to accept them and overcome them.”*	Participant 6: 39-year-old woman, living with her spouse, no children, no professional activities Mucoviscidosis and heart–lung transplant (Massage, singing)
5	« Je crois que je suis plus acceptante quoi, d’une certaine manière… que la maladie progresse avec une part d’incertitude.»	*"I think I'm more accepting, in a way… that the disease is progressing with some uncertainty."*	Participant 17: 54-year-old woman living with her spouse and children, no professional activities HIV (Meditation, singing)
6	« Le théâtre du vécu a changé ma perception de l’avenir. Oui, je me suis dit c’est possible d’être bien, heureux, tel qu’on est… Ce ne sont pas les mêmes, mais il y a d’autres possibles.»	*“The Theatre of life* [one of the available CTs]* has changed my perception of the future. Yes, I told myself, it's possible to be well, happy, just as we are… They are not the same, but there are other possibilities”*	Participant 139: 52-year-old woman living alone with children, administrative assistant Primary dilated cardiomyopathy (Massage, theatre of life)
7	« L’art-thérapie, je dirais que ça m’a fait plus réfléchir sur moi-même, sur qui je suis et qui je veux être. Qui je veux être, voilà. Disons que j’ai moins peur de me montrer comme je suis, je me cache moins derrière une image, derrière une façade.»	*"Art therapy, I would say, made me think about myself more often, who I am and who I want to be. Who I want to be, that’s it. Let's say that I am less afraid of showing myself the way I am, I am less hiding behind an image, behind a facade.”*	Participant 15: 48-year-old woman living with her spouse and children, beautician Low back pain, endometriosis (Shiatsu, Art therapy)
8	« En fait ça m’a vraiment beaucoup aidée, le massage, à trouver des réponses à des questions et surtout savoir quelle route emprunter quoi… pour le travail, là, si j’ai postulé… enfin j’ai postulé de moi-même, mais en sortant d’une séance, j’ai renvoyé un petit texto à la personne qui s’occupait du recrutement… J’ai pris des actions très concrètes à l’issue de ces rendez-vous, de ces massages…»	*"In fact, it really helped me a lot, the massage, to find answers to some questions and especially to know which road to take… for example about work, I wanted to find one, well, I applied by myself, right after a session … I took very concrete actions after these massages …"*	Participant 84: 30-year-old woman living with her spouse and children, higher intellectual profession Rheumatoid arthritis (Massage, Art therapy)
9	« Quand je sens que j’ai des idées noires. Et quand tout d’un coup, je me mets à penser à ma respiration, et donc à me focaliser sur quelque chose qui est stable, et bien là, mais tout de suite, je sens que tout s’apaise. Mais c’est extraordinaire en fait»	*"When I have dark thoughts, then all of a sudden, I start thinking about my breathing, and therefore focus on something that is stable, there, right away, I feel that everything is calming down. It's wonderful, actually.”*	Participant 10: 42-year-old woman, living with spouse and children, higher intellectual profession Mucoviscidosis (Meditation, writing)
10	« Et au théâtre du vécu, j’ai pu aussi l’exprimer par une scène… voilà, il y avait l’inceste. Ça m’a fait ressortir ça. Et donc, ça m’a beaucoup bousculée, beaucoup, beaucoup. Et je pense qu’il y a quelque chose qui a été libéré. Soulagée et libérée. J’ai pu mettre des mots quoi… C’était là et il fallait que ça sorte.»	*"And in the theatre of life, I was able to express it through a scene… there was incest. It brought that out of me. And so, it pushed me a lot, a lot, a lot. And I think there's something that was released. Relieved and liberated. I was able to put into words what… It was there and it had to come out.”*	Participant 3: 62-year-old woman living with her spouse and children, retired Anal incontinence (Massage, theatre)
11	« Et la famille, c’est pareil, je peux mieux me positionner, dire les choses, faire l’effort de dire les choses, voilà. Avant, je ne disais pas « parce que ça ne va pas lui plaire», ben tant pis, maintenant, je le dis quand même. Ça me prend du temps, mais j’y arrive»	*"And within my family, it’s the same, I am more able to take a stance, say things, make the effort to say things, and that's it. Before, I didn't speak because [people were] not going to like it, but now I speak anyway. It takes a long time, but I'm getting there.”*	Participant 74: 67-year-old woman living alone, retired Breast cancer, hereditary angle-closure glaucoma, age-related macular degeneration, osteoarthritis (Meditation, art therapy)

#### Effects on impulse

In the DERS, Impulse focuses on difficulties to control oneself in a negative emotional context. Half of the interviewees (51%, 57/112) described a better control of their impulsive behaviours (verbatim 1).

#### Effects on awareness

In the DERS, awareness assesses the extent to which individuals are able to monitor one’s emotions. Half of the participants (51%, 57/112) reported an improvement in their ability to listen to their feelings, to better perceive and identify their emotions (verbatim 2). Furthermore, the majority of the participants (66%, 74/112) described a better ability to analyse sensations, thoughts, issues and self-limiting behaviours (verbatim 3).

#### Effects on clarity

Clarity assesses an individual’s ability to identify, distinguish and describe specific emotion. Nearly half of the participants (47% of the interviewees, 53/112) reported a better understanding of their emotions and how they impacted on their physical symptoms (verbatim 4).

#### Effects on acceptance

Acceptance is focused on an individual’s ability to accept one's emotional response. Only 12% (14/112) of them spontaneously reported a better acceptance of their emotions after 6 months of intervention. However, the majority of the participants (69%, 77/112) described a better acceptance of their difficulties and chronic illnesses (verbatim 5).

#### Effects on strategy

Strategy assesses an individual’s ability to implement emotional regulation strategies in a negative emotional context. A large majority of participants (87%, 98/112) reported having implemented new emotional regulation strategies. We identified either cognitive (77%, 86/112) or behavioural strategies (74%, 83/112).

Among the most quoted cognitive strategies was the ability to put things into perspectives, with a greater focus on positive aspects of their life (51%, 58/112). The participants described a more positive relationship to their body and disease, but also a better future outlook (68%, 77/112) (verbatim 6). Most of the participants (84%, 94/112) reported an increased self-awareness of their aspirations, deep needs, values and capacities for action (verbatim 7). Regarding engagement in new behaviours, several participants (38%, 42/112) talked about making important decisions to meet their deepest needs and aspirations (verbatim 8).

Among the behavioural strategies, breathing techniques were used by the majority of the participants (58%, 65/112). The participants also kept using in their daily life techniques learned during the interventions: meditation, yoga, singing, self-massages, Pilates, drawing, and writing (verbatim 9). Another new strategy implemented by the patients was emotion expression. For example, 69% (77/112) of the participants allowed themselves to feel and express their emotions more often, without repressing them, in particular by letting themselves cry. During the 6 months that lasted the interventions, 35% (39/112) of the participants were able to release previously repressed emotions, often related to past psycho-traumatic life events (verbatim 10). Finally, 57% of the participants (64/112) reported new relational strategies by being more assertive, communicating their emotions better and expressing their needs or disagreements (verbatim 11).

## Discussion

Our study is the first to evaluate the effect of MBATs on emotional regulation in chronically ill patients by use of a randomized clinical trial design. Our main quantitative analysis was inconclusive as it did not show a favourable effect of MBATs on emotional regulation, compared to the control intervention, as measured by the DERS questionnaire. However, a statistically significant difference was observed between participants who completed at least 80% of their activity program and the control group. Conversely, the qualitative analysis supported a positive effect of MBATs on emotional regulation: nearly half of the participants reported an improvement in their impulsiveness, emotional awareness and emotional clarity. Moreover, most of the participants reported having implemented new emotional regulation strategies.

Regarding qualitative data, as no comparisons were planned between groups, every interviewee that had participated in MBAT interventions was interviewed and no patient from control group was. It is thus impossible to differentiate between our program’s effectiveness and the natural evolution of chronically ill patients’ emotional skills in a qualitative manner. Only the comparison between the qualitative and quantitative results in the intervention group was conducted and the difference in results observed between both approach need to be discussed.

Several hypotheses can be put forward to explain these discrepancies. First, regarding our quantitative results, the intervention could have had a real effect on emotional regulation in participants, adequately measured but limited in size. Based on the existing literature, our study was designed to detect a between-group difference of 19 points [31], and we only observed a change of 4.8 points. However, in the case that this result is not merely due to random variation, the clinical impact of such a small change remains debatable.

Second, this apparent paradox could be explained by changes that may have not been detected by the DERS. This could be due to limitations of the tool itself, or by the fact that the improvements reported by the participants only limitedly correspond to the emotional regulation model proposed by Gratz and Romer [9], on which the DERS is based. Other emotional regulation measures exist, based on different framework (e.g. Emotion Regulation Questionnaire, based on Gross conceptual framework [34]) and have shown that they may capture relatively distinct aspects of ER from those measured by the DERS [35]. Moreover, based on the qualitative analysis, broader constructs, such as that of recovery in the field of mental health, may have more appropriately measured the effect of MBATs on chronically ill patients. Indeed, personal recovery encompasses several related notions such connectedness, hope and optimism about the future, identity, meaning in life, and empowerment [36] Recovery, however, is only suited to mental health patients and no equivalent exists, to our knowledge, for somatic diseases. An in-depth qualitative analysis of the participants’ interviews is underway to better identify the dimensions impacted by our intervention.

Third, these discrepancies could be a result of effect dilution. Indeed, our intervention strategy relied on various MBATs and was applied to a rather heterogeneous study population. Indeed, the choice of this study population and the variety of activities was decided according to the pragmatic nature of the trial, which aimed at reproducing the conditions in which these interventions were already offered to chronically ill patients in Nantes within the FCS. However, the cost of this flexibility could have been paid on different aspects of the study. First, the heterogeneity of the studied population (pathologies’ type and duration, for example) may have contributed to a highly variable impact on patients’ emotional regulation. Some might have experienced an important benefit from it while others didn’t, partly due to the lack of adaptation of the intervention to their specific needs. On the other hand, the multiplication of interventions led to a multiplication of instructors, whose ability to elicit engagement amongst participants regarding the proposed content may have been unequal. All these elements may have led to the pooling of various effects of the different MBATs on various patient profiles, and thus turning effect of a particularly efficient MBAT on a particular clinical context indistinguishable, as our sample size did not permit stratification on each MBAT.

When we limited our analysis to highly adherent patients, we observed a stronger and significant effect. This observation in the most adherent population is coherent with the elements described above, as the most adherent patients may have been the patients which found the best match between their personal situation, the proposed MBATs and the personality of their instructors, therefore boosting their will to pursue the intervention program to its end and get the most out of it. Unfortunately, a posteriori analysis revealed that the adherent participants did not differ from the rest of our sample, ruling out the possibility of detecting early on who could benefit the most of our intervention strategy.

Finally, several limitations that could have had an impact on our results must be noted. First of all, we evaluated the effect of our MBT-based intervention after 6 months, which could be too short for developing significant changes in emotional regulation. Therefore, the participants may have notice small and encouraging changes that couldn’t yet have a sizeable effect that could be measured by the DERS. Another limitation is that the qualitative methodology involved undirected interviews, which means that the interviewers did not specifically question the participants on the dimensions identified in the DERS. Only the dimensions in which the participants observed or perceived major changes, were thus covered by the qualitative analysis. A last limitation to be noted is the subjectivity of one of the qualitative analysts, which was deeply involved in the diffusion and structuration of MBATs in our local setting. Despite efforts to adopt a neutral stance, this positive bias towards MBATs should be acknowledged.

Some encouraging evidence is available regarding the effectiveness of MBT in improving some components involved in emotional regulation or similar concepts. A mapping review reported that art therapy interventions in cancer patients improved emotional condition [37]. Theatre is also used by psychiatrists to decrease the suppression of the emotion [15]. Meditation improved acceptance of stressors and reduced distress and rumination and focused attention led to greater self-regulation abilities [38]. Finally, in a randomized trial involving 60 patients, 8 weeks of practice of a combination of aerobic fitness and mindfulness based yoga improved implicit emotion regulation [39].

To our knowledge, our study is the first to assess impact of MBATs on emotional regulation as assessed by the DERS in a population of chronically ill patients. Existing research is mainly focused on the evaluation of alternatives to MBT in improving emotional regulation, for example Emotion Regulation Group Therapy, Acceptance and Commitment Therapy, Dialectical Behaviour Therapy and Cognitive Behavioral Therapy, showing evidences regarding their capacity to significantly improve emotional regulation ability [40].

## Conclusion

The quantitative results of this study did not support the causal effect of MBATs on patients' emotional regulation. However, the qualitative part of our mixed methods study highlighted a wild range of potential benefits of these complementary therapies in the population of chronically ill patients regarding their ability to regulate their difficulties. Further research using larger sample sizes, issued from a more homogeneous population, testing a restricted set of MBATs with experienced trainers is needed to fully state on MBATs’ benefits regarding emotional regulation.

### Supplementary Information

**Additional file 1.** Analysis of pragmatism of the EVAD Trial with the PRECIS-2 tool.

**Additional file 2.** Description of DERS sub-scores (% of responses).

**Additional file 3.** Dataset.

## Data Availability

Data are available in supplementary materials (Additional file 3). If further material is needed, please contact the corresponding author.
