# Supplementary material for: Mind–body and art therapies impact on emotional regulation in patients with chronic diseases: a pragmatic mixed-methods randomized controlled trial
Source: BMC Complement Med Ther. 2023 Sep 28;23:344. doi: 10.1186/s12906-023-04173-8 (PMC10536705; doi:10.1186/s12906-023-04173-8)

Additional file 1. Analysis of pragmatism of the EVAD Trial with the PRECIS-2 tool


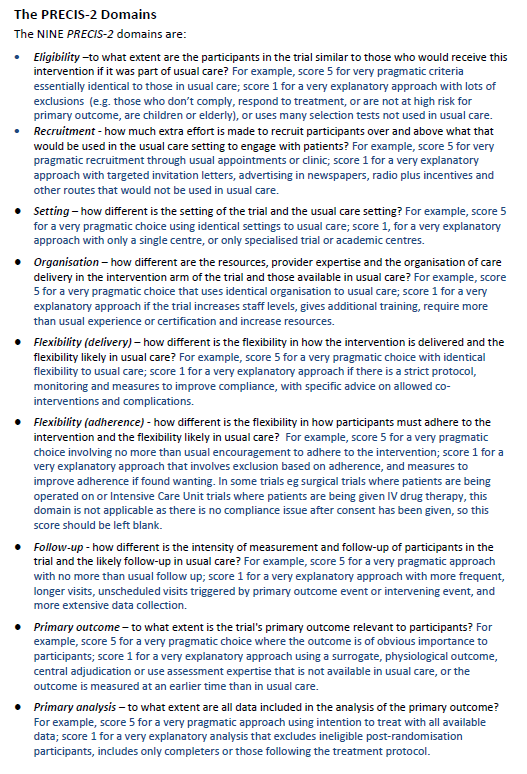


| Domain | Definition | Rated | Explanation |
| --- | --- | --- | --- |
| Eligibility Criteria | Who is selected in the trial ? | 5 | Population studied is the population handled in usual care |
| Recruitement path | How are participant recruited in the trial ? | 4 | Advertising was done for the study in the press, but most of patients comes from usual care |
| Setting | Where is the trial being done ? | 5 | The trial was done in the usual care setting |
| Organisation | What expertise and ressources are needed to deliver the intervention ? | 4 | No expertise was needed other than available in usual care. However, for coordination of study purpose, supplementary human resources were available for the time of the study |
| Flexibility - delivery | How should the intervention be delivered ? | 5 | Real life flexibility, with high level of adaptability of the intervention to patients needs |
| Flexibility - adherence | What measures are in place to make sure participants adhere to the intervention ? | 3 | Presence in each sessions was monitored, and patients were contacted by telephone *a posteriori* in case of absence. |
| Follow-up | How closely are participant followed-up ? | 3 | Patients were followed by dedicated research staff and meet with the team more than needed by usual care |
| Primary outcome | How relevant is it to participants ? | 4 | Using a score as a primary outcome may be limited to capture complexity of the intervention effect. However, use of qualitative investigation may have mitigated this point. |
| Primary analysis | To what extent are all data included ? | 5 | Intention to treat analysis was performed. |

Analysis of EVAD Trial regarding the nine domains


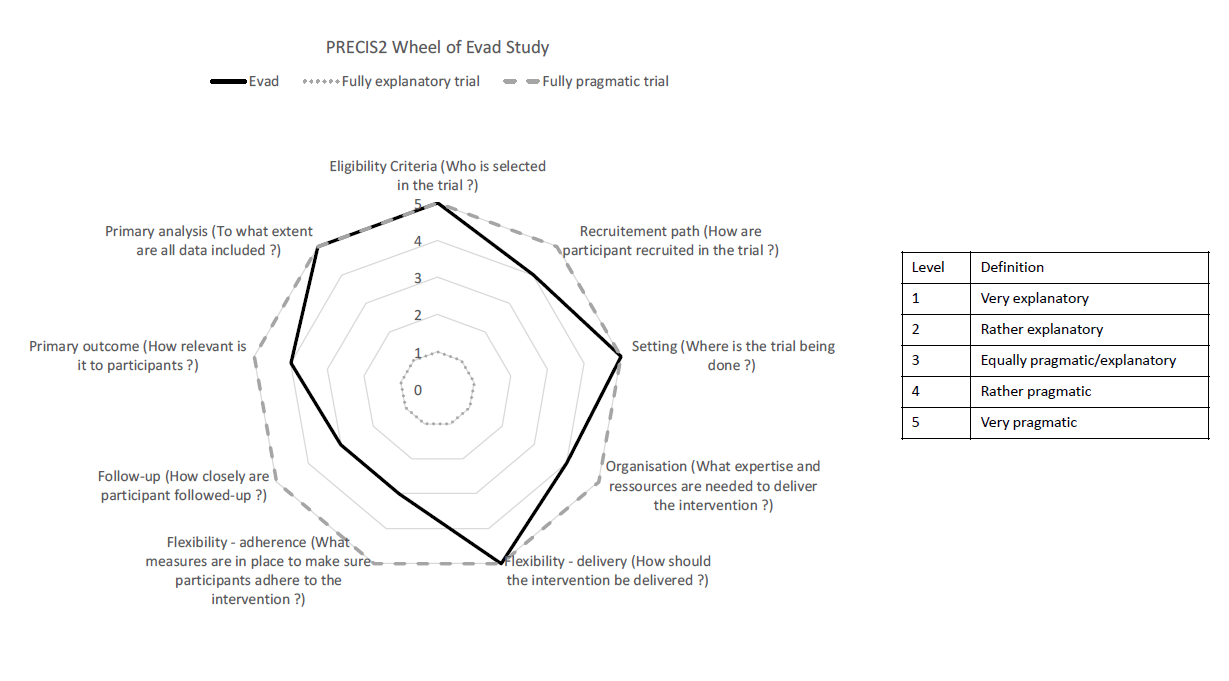

Supplement: Supplementary file 1 — Additional file 1. Analysis of pragmatism of the EVAD Trial with the PRECIS-2 tool. [file 12906_2023_4173_MOESM1_ESM.docx]
