# Supplementary material for: Mind–body and art therapies impact on emotional regulation in patients with chronic diseases: a pragmatic mixed-methods randomized controlled trial
Source: BMC Complement Med Ther. 2023 Sep 28;23:344. doi: 10.1186/s12906-023-04173-8 (PMC10536705; doi:10.1186/s12906-023-04173-8)
Supplement: Supplementary file 2 — Additional file 2. Description of DERS sub-scores (% of responses). [file 12906_2023_4173_MOESM2_ESM.docx]

Additional file 2: Description of DERS sub-scores (% of responses)

| *Non-Acceptance of Emotional Responses (Non-Acceptance)* | Almost never | Sometimes | About half the time | Most of the time | Almost always | No response |
| --- | --- | --- | --- | --- | --- | --- |
| 11. When I’m upset, I become angry with myself for feeling that way | 17.4 | 29.5 | 14.1 | 24.8 | 14.1 | 0.7 |
| 12. When I’m upset, I become embarrassed for feeling that way | 16 | 36 | 15.3 | 20.7 | 12 | 0 |
| 21. When I'm upset, I feel ashamed with myself for feeling that way | 33.6 | 38.9 | 9.4 | 8.7 | 9.4 | 0.7 |
| 23. When I'm upset, I feel like I am weak | 23.5 | 49.7 | 8.7 | 12.1 | 6 | 0.7 |
| 25. When I'm upset, I feel guilty for feeling that way | 31.3 | 40 | 9.3 | 13.3 | 6 | 0 |
| 29. When I'm upset, I become irritated with myself for feeling that way | 11.4 | 44.3 | 12.1 | 20.8 | 11.4 | 0.7 |
| Difficulties Engaging in Goal-Directed Behaviour (Goal) | **Almost never** | **Sometimes** | **About half the time** | **Most of the time** | **Almost always** | **No response** |
| 13. When I’m upset, I have difficulty getting work done | 14.3 | 34 | 15.6 | 27.2 | 8.8 | 2 |
| 18. When I'm upset, I have difficulty focusing on other things | 2.7 | 28.7 | 22 | 28 | 18.7 | 0 |
| 20. When I'm upset, I can still get things done | 2.7 | 10.7 | 19.3 | 47.3 | 20 | 0 |
| 26. When I'm upset, I have difficulty concentrating | 10 | 34 | 24 | 20 | 12 | 0 |
| 33. When I'm upset, I have difficulty thinking about anything else | 8 | 40.7 | 20 | 21.3 | 10 | 0 |
| Impulse Control Difficulties (Impulse) | **Almost never** | **Sometimes** | **About half the time** | **Most of the time** | **Almost always** | **No response** |
| 3. I experience my emotions as overwhelming and out of control | 11.4 | 43 | 17.4 | 12.1 | 16.1 | 0.7 |
| 14. When I’m upset, I become out of control | 57.3 | 24 | 6.7 | 7.3 | 4.7 | 0 |
| 19. When I'm upset, I feel out of control | 41.6 | 34.9 | 10.1 | 6.7 | 6.7 | 0.7 |
| 24. When I'm upset, I feel like I can remain in control of my behaviours | 10.1 | 18.2 | 18.2 | 35.1 | 18.2 | 1.3 |
| 27. When I'm upset, I have difficulty controlling my behaviours | 32.2 | 43.6 | 12.1 | 8.7 | 3.4 | 0.7 |
| 32. When I'm upset, I lose control over my behaviours | 47.7 | 35.6 | 8.1 | 6.7 | 2 | 0.7 |
| Lack of Emotional Awareness (Awareness) | **Almost never** | **Sometimes** | **About half the time** | **Most of the time** | **Almost always** | **No response** |
| 2. I pay attention to how I feel | 4.7 | 9.3 | 19.3 | 42 | 24.7 | 0 |
| 6. I am attentive to my feelings | 5.4 | 11.4 | 18.8 | 38.3 | 26.2 | 0.7 |
| 8. I care about what I am feeling | 4.7 | 8.7 | 10.7 | 44.7 | 31.3 | 0 |
| 10. When I’m upset, I acknowledge my emotions | 2 | 5.3 | 4.7 | 34.7 | 53.3 | 0 |
| 17. When I'm upset, I believe that my feelings are valid and important | 4 | 22.1 | 14.8 | 43 | 16.1 | 0.7 |
| 34. When I'm upset, I take time to figure out what I'm really feeling | 8.1 | 21.5 | 20.8 | 34.9 | 14.8 | 0.7 |
| Limited Access to emotional regulation Strategies (Strategy) | **Almost never** | **Sometimes** | **About half the time** | **Most of the time** | **Almost always** | **No responsev** |
| 15. When I'm upset, I believe that I will remain that way for a long time | 42.6 | 29.7 | 12.8 | 10.8 | 4.1 | 1.3 |
| 16. When I'm upset, I believe that I'll end up feeling very depressed | 53.7 | 26.8 | 8.7 | 8.7 | 2 | 0.7 |
| 22. When I'm upset, I know that I can find a way to eventually feel better | 4.7 | 16.1 | 16.8 | 36.9 | 25.5 | 0.7 |
| 28. When I'm upset, I believe there is nothing I can do to make myself feel better | 43.6 | 35.6 | 9.4 | 8.1 | 3.4 | 0.7 |
| 30. When I'm upset, I start to feel very bad about myself | 24.2 | 44.3 | 13.4 | 8.7 | 9.4 | 0.7 |
| 31. When I'm upset, I believe that wallowing in it is all I can do | 70 | 19.3 | 3.3 | 5.3 | 2 | 0 |
| 35. When I'm upset, it takes me a long time to feel better | 5.3 | 38.7 | 26 | 22 | 8 | 0 |
| 36. When I'm upset, my emotions feel overwhelming | 11.3 | 40.7 | 16.7 | 18.7 | 12.7 | 0 |
| Lack of Emotional Clarity (Clarity) | **Almost never** | **Sometimes** | **About half the time** | **Most of the time** | **Almost always** | **No response** |
| 1. I am clear about my feelings | 1.3 | 8.1 | 13.4 | 55 | 22.1 | 0.7 |
| 4. I have no idea how I am feeling | 59.7 | 25.5 | 5.4 | 6.7 | 2.7 | 0.7 |
| 5. I have difficulty making sense out of my feelings | 32.4 | 44.6 | 12.2 | 9.5 | 1.4 | 1.3 |
| 7. I know exactly how I am feeling | 2 | 13.3 | 26 | 41.3 | 17.3 | 0 |
| 9. I am confused about how I feel | 38.3 | 43.6 | 8.1 | 6 | 4 | 0.7 |
